# Hibernating astronauts—science or fiction?

**DOI:** 10.1007/s00424-018-2244-7

**Published:** 2018-12-19

**Authors:** A. Choukèr, Jürgen Bereiter-Hahn, D. Singer, G. Heldmaier

**Affiliations:** 1 Laboratory “Translational Research Stress and Immunity”, Department of Anesthesiology, Hospital of the University of Munich, Marchioninistraße 15, 81377 Munich, Germany; 2 Institute for Cell Biology and Neurosciences, Goethe University Frankfurt, Max-von-Lauestrasse 13, 60439 Frankfurt am Main, Germany; 3 Division of Neonatology and Pediatric Critical Care Medicine, University Children’s Hospital, University Medical Center Hamburg-Eppendorf, Martinistraße 52, 20246 Hamburg, Germany; 4 Animal Physiology, Faculty of Biology, Marburg University, Karl-von-Frisch-Straße 8, 35043 Marburg, Germany

**Keywords:** Hibernation, Torpor, Metabolism, Spaceflight

## Abstract

For long-duration manned space missions to Mars and beyond, reduction of astronaut metabolism by torpor, the metabolic state during hibernation of animals, would be a game changer: Water and food intake could be reduced by up to 75% and thus reducing payload of the spacecraft. Metabolic rate reduction in natural torpor is linked to profound changes in biochemical processes, i.e., shift from glycolysis to lipolysis and ketone utilization, intensive but reversible alterations in organs like the brain and kidney, and in heart rate control via Ca^2+^. This state would prevent degenerative processes due to organ disuse and increase resistance against radiation defects. Neuro-endocrine factors have been identified as main targets to induce torpor although the exact mechanisms are not known yet. The widespread occurrence of torpor in mammals and examples of human hypometabolic states support the idea of human torpor and its beneficial applications in medicine and space exploration.

## Background

For thousands of years, humans dream to fly to the stars. Since some hundred years ago, the distances which we have to pass just to reach the moon or any other planet in our solar system became clear. The fascination of space exploration was even stimulated by this knowledge. Mars is a preferred destination for exploration first by robotic inspection yielding basic knowledge followed by human presence for in-depth investigations. Keeping astronauts mentally and physically in good shape on such a journey of about 560-day duration is one of the great challenges in manned space flights. Flying in a small capsule combines confinement with low physical exercise. This can cause severe mental problems [78], alter metabolism [92], and influence the immune system, as was shown in bed rest studies and investigations on the effects of confinement, e.g., during the MARS500 study, and in Antarctic stations and underwater habitats [78]. Nutrition, waste management, and environment control have to be tackled. Reduction of nutritional requirements and a state of reduced awareness during the time of flight attaining a metabolic state called *torpor* (*torpeo* lat. = to be stiff, numb; corresponding to the physiological situation in hibernating animals during hibernation) would be a game changer in solving these problems for space exploration [37].

## Torpor versus hypothermia, coma, and sleep

Torpor is the physiological basis for hibernation. During torpor, many physiological activities are reduced resulting in decrease of metabolism, body temperature, heart rate, and respiration to a fraction of their normal rate [25, 65, 113]. Many small mammals like bats, mice, or lemurs become torpid for several hours per day (less than 24 h) during their circadian resting phase, a behavior which is called daily torpor. Hibernation is a seasonal behavior consisting of series of multiday torpor bouts (i.e., individual torpor bouts greater than 24 h, most often 10–20 days) interrupted by arousals with euthermic periods lasting 1–2 days. Hibernation is found in many rodent species, bats, lemurs, marsupials, hedgehogs, and bears [33, 43]. As an exception, black bears do not interrupt hibernation by arousals but hibernate with depressed metabolism at a body temperature > 30 °C [106, 114]. A similar observation was made in lemurs hibernating in thin-walled tree holes where body temperature was raised to 30 °C every day, whereas lemurs in thick-walled trunks hibernated with lower body temperatures and showed repeated arousals like all other hibernators [22]. This suggests that arousals may be linked to the occurrence of very low body temperatures in hibernation.

Although “human hibernation” is often confused with clinical hypothermia, these are completely different states of metabolic reduction: Whereas hypothermia refers to a state of “passive cooling with subsequent reduction in metabolic rate in critical care patients under drug-induced suppression of thermoregulation,” torpor reflects a state of “endogenous metabolic reduction with subsequent fast and extensive decrease in body temperature being limited to a minimal level by maintained thermoregulation” [43, 95, 96]. Thus, long-lasting metabolic reduction in humans might not necessarily require forced external cooling, but to elicit a state of endogenous hypometabolism would induce a much more efficient reduction of metabolic rate (MR) than is possible by hypothermia [33].

Coma is a state of loss of consciousness caused either by cerebral trauma (direct or indirect) or neurologic injuries (e.g., stroke), drugs, or severe metabolic disturbances (e.g., diabetic coma, uremic coma). Such a condition is not comparable to torpid states as the MR is only severely affected in the damaged brain areas, while overall a sympathetic predominance is observed proportionally to the “depth” of the coma. It is not related to reduced MR [66].

The relation between torpor and sleep, especially the question if there is a causal relationship between sleep and torpor, remains still an unresolved issue. During interbout arousals of ground squirrels, an extension of slow-wave sleep is observed, which typically follow periods after sleep deprivation. The conclusion of this behavior was that squirrels aroused from torpor to sleep (“Warming up for sleep?”), indicating that a sleep debt has been built up during torpor which has to be compensated by an extended period of slow-wave sleep [20, 107]. Later studies questioned this conclusion because forced suppression of sleep during the euthermic period did not prevent the onset of the next multiday torpor bout [55]. Nevertheless, slow-wave sleep is regularly observed following arousal from multiday torpor bouts in hibernators as well as following arousal from short daily torpor bouts in hamsters and mice [111, 112]. A link between sleep and torpor is further supported by the 24-h rhythm of daily torpor in hamsters, bats, and mice since torpor occurs preferably during the sleeping phase of the 24-h rhythm [7, 47, 54, 77].

## Metabolic rate reduction in torpid mammals and how we can predict metabolic rate in humans during torpor

**Fig. 1:**
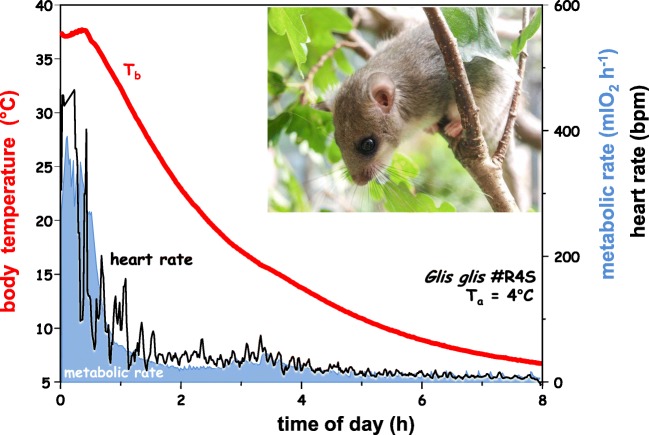
Spontaneous entrance into a hibernation bout in a dormouse (*Glis glis*) at 5 °C ambient temperature. Entrance is characterized by a rapid and synchronous depression of metabolic rate, ventilation, and heart rate. Body temperature gradually approaches the level of ambient temperature (modified from [43, 100])

Torpor is characterized by a reduction of the MR (measured as oxygen consumption), heart rate, or ventilation frequency accompanied by a controlled decrease of body temperature (Fig. 1). In small mammals, body temperature may closely approach ambient temperature but in larger mammals it may be reduced by a few degrees only. In torpor, most body functions are suppressed to a fraction of their normal rate and metabolic pathways become reorganized in a manner specific for each organ, i.e., shift from glycolysis to lipolysis [1, 42]. As a consequence, this shift increased ketone-based supply for the brain and the heart, respectively [2]. Furthermore, mitochondrial respiration is reduced, as well as gene expression, protein synthesis, and protein degradation are largely suppressed [9, 10, 26, 110, 114].

**Fig. 2:**
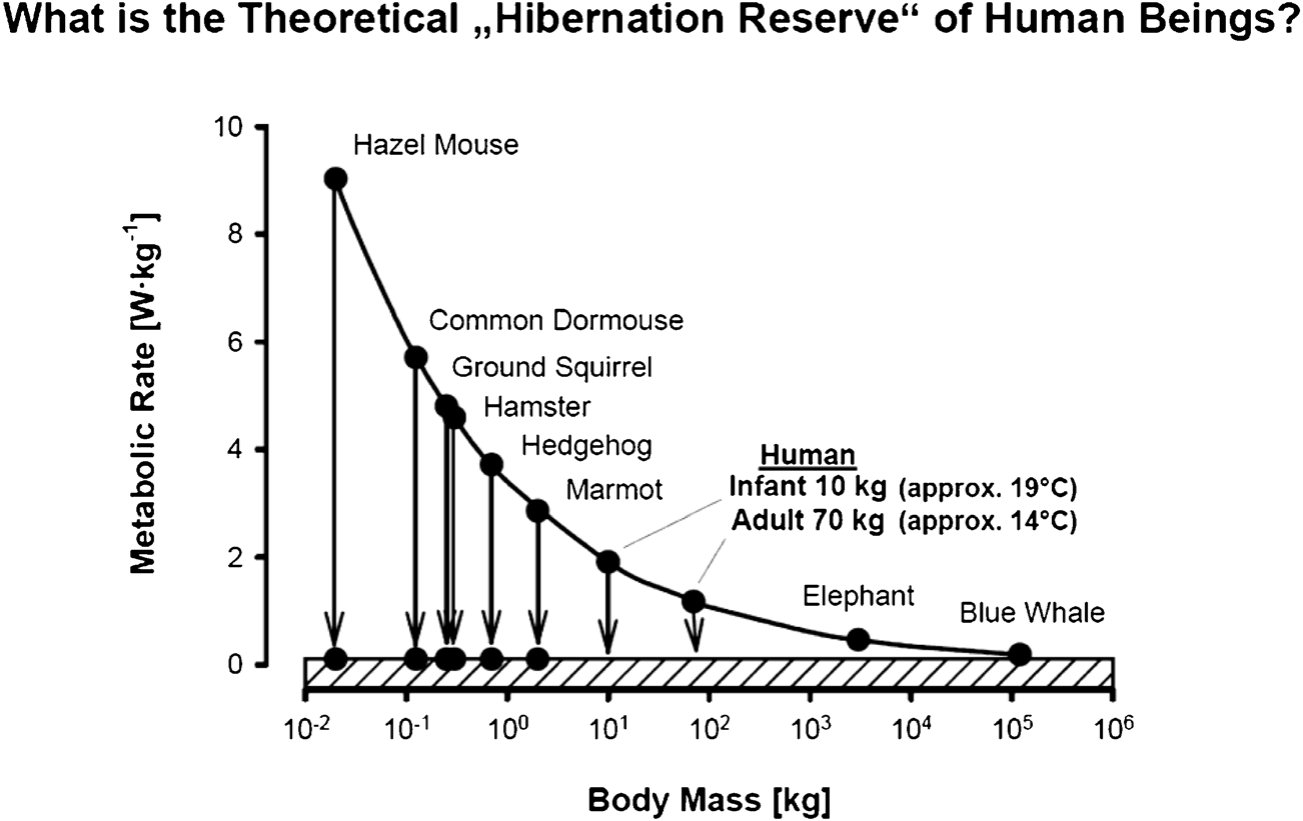
MR and body mass are allometrically related to each other, i.e., the MR of animals with high body mass is relatively lower than that of animals with low body mass. According to this relation, the minimum MR reached by very large mammals (hatched area) also defines the minimum MR in torpor. The difference between the euthermic MR and minimum MR in torpor (arrows) determines the MR reduction which is achievable by torpor (arrows) (from [94])

Comparative physiological investigations have shown that hibernating mammals exhibit a more or less uniform mass-specific MR, amounting to some 0.1 W/kg. This is true for both small hibernators and denning black bears although the latter exhibit a much smaller decrease in body temperature [43, 106]. In other words, the “mouse-to-elephant” curve (metabolic size allometry, Kleiber’s rule), reflecting the overall decrease in specific basal MR with increasing body mass, seems to be “switched off” during hibernation (Fig. 2). Remarkably, the minimal metabolic rate of hibernators (0.1 W/kg) corresponds to the mass-specific basal metabolic rate that is attained by the very largest mammals, such as whales by pure body size, and might thus indicate a common lower limit to metabolic activity among mammals. Given the fact that the mass-specific basal MR of human beings amounts to some 1.0 W/kg, there is a theoretical 90% interspace left for MR reduction in humans from a strictly comparative physiological point of view [90, 94].

## Protective effects of hypometabolism

Hypometabolism by natural torpor in humans could have a strong impact on medicine in critical situations [6, 12]. This can apply for instance for transplantation surgery, as hibernators show resilience to kidney injury which can guide for new measures to preserve explanted human kidneys for transplantation and to hereby increase the organ function in the recipient [86]. Moreover, it has been summarized how research in larger mammals may help to understand and hereby prevent unfavorable consequences e.g. of immobilization, e.g., the bear has mitigated those health challenges humans face as well, e.g., muscle wasting and osteoporosis. Both constitute a severe problem in bed-rested or partly immobilized patients [3]. The knowledge derived in the last two decades from research on hibernators and their hypoxic tolerance [29, 56] will help to understand and hereby modulate regulatory mechanisms to prevent the hypoxemic injuries of the entire body (shock) or of single organs (e.g., brain, liver), thus increasing hypoxia tolerance and avoiding unfavorable conditions of tissue perfusion in the course of (organ) shock and inflammation. Moreover, this knowledge on metabolic regulations in hypoxic tissues will also help to better understand the dynamics of tumor growth, and can shed new light on vulnerable therapeutic windows. In addition, if a tumor could be put into a state of quiescence, this would gain time to design an adequate anti-cancer treatment [64, 74].

The induction of a moderate state of hypothermia is common in clinical practice. Body temperature is cooled to 35 °C or even 32 °C in order to slow down metabolism and to preserve and extend the homeostasis in those cells that can recover from damages in the course of trauma or anoxia, or organ transplantation. The most oxygen and metabolically sensitive organ is the brain, representing about 2% of body weight but consuming 20% of oxygen [51]. Interestingly, however, all studies and meta-analyses have not yet provided evidence that cooling is beneficial for the brain, i.e., to improve defined outcome parameters when (i) hypothermia was used in the course of the treatment of brain injury. While results from some trials and experimental approaches seem very promising, to date, there remains a lack of “high-quality evidence that hypothermia is beneficial in the treatment” of patients with a traumatic brain injury [59] nor was there proven clinical evidence that (ii) induced hypothermia during neurosurgery would have been linked with a significant reduction in mortality or patients’ neurological disability [30, 60]. The protective effect of hypothermia (in terms of preventing the brain from hypoxic–ischemic damage) appears to be limited by the moderate degree of metabolic reduction that is attainable in the clinical setting. Moreover, the depth of cooling is limited by the adverse effects of reduced temperatures on several body functions. Therefore, an alternative protective strategy allowing an endogenous metabolic reduction independently of external cooling could be of great benefit in critical care medicine.

This lack of a stabilizing effect of induced hypothermia against tissue damage may not entirely reflect the conditions of such induction in healthy tissues. It indicates though that cooling is not equivalent to inducing a reduction of metabolism. In contrast, during natural torpor, the extent of hypometabolism is much larger than the effect of hypothermia [33, 76, 106]. Physiological processes are well controlled in natural torpor and can be sustained for a prolonged period of time. The use of this pathway to hypometabolism would offer a wide range of benefits in clinical practice as compared to artificial hypothermia, and will range from the acute prevention of metabolism deficiency in severely compromised and hypoxemic states (e.g., after trauma or asphyxia) to energy preservation in organs prepared for transplantation. Moreover, controlling cell energy turnover can not only help to control obesity and aging [117] processes, but also will allow limiting destructive catabolic states in severe illnesses such as sepsis and immobilization. Despite inactivity and starvation, hibernating animals do not suffer from atrophy of bone or musculoskeletal mass and strength [50, 68, 88], to an extent which would be expected by disuse during several months of physical rest in their hibernaculum, or in humans during months of bed rest [17, 88]. Interestingly, this might depend though on the size of the animal as some microstructural bone loss was observed in 13-lined ground squirrels during hibernation while this was not observed in larger hibernating mammals [40, 69]. Thus, torpor strongly preserves organ and tissue integrity. This phenomenon could also be relevant during long spaceflights to counteract the effects of microgravity on disuse-related muscle and bone degeneration.

Organs which undergo severe changes during torpor and in hibernation, e.g., the intestine, lung, kidney, and also brain, recover very fast after arousal. For example, *lung* tissue in Syrian hamsters undergoes strong reorganization from torpor to the euthermic state: Autophagy highly increases in late torpor and during arousal most probably because of mTOR activation [105]. Reduced apoptosis and necrosis immediately after arousal from hibernation are hypothesized to underlie better maintenance of epithelial/endothelial barrier function [28]. In addition to anti-apoptotic proteins, potential molecular mechanisms that serve to resist tissue degradation during hibernation include activated serine/threonine kinase Akt [28] and the redox-sensitive transcription factor NF-κB [11]. More recently, increased formation of endogenous H_2_S, e.g., by induction of cystathionine-beta-synthase (CBS) expression during late torpor and early arousal, has been implicated as a mechanism protecting cells of hibernators from damage [19, 103, 104], e.g., increased renal expression of CBS reduces apoptosis and by this prevents kidney damage as it occurs in rats after rewarming from deep hypothermia [24].

The *brain* undergoes major remodeling in the torpid state during hibernation, i.e., reduction in number and height of synaptic boutons, and hyperphosphorylation of the neuroskeletal protein tau. These changes are rapidly reversed during arousals [23, 67, 83, 99], protecting the brain from loss of memory [16, 89, 115]. Elucidation of these processes could have strong impact on treatment of neurodegenerative diseases [82].

The *heart* plays a crucial role in the regulation of body homeostasis and circulation at all stages of torpor and is a priori highly susceptible to tissue and cellular damages. But during hypometabolic states in hibernation, either the large (bear) or smaller (squirrels) animals show sustained cardiac function—as measured by its output—reduced by ~ 75% (bears) or by ~ 97% (squirrels) [76]. In relation to animal size and core body temperature of the hibernating animal, frequency of the heart beat decreases, but remains at a sufficient and constant rate although the body temperature can be low (< 10 °C). This is related to a “reduced Ca^2+^ entry into the cell and concomitant enhancement of Ca^2+^ release from and reuptake by the sarcoplasmic reticulum” as Horii et al. [48] reviewed. A very recent controlled study on European ground squirrels quantified protein upregulations of antioxidant defense enzymes (e.g., manganese and copper/zinc superoxide dismutases) in the myocardium. Those hibernation-related phenotypes of the myocardium are hence characterized by changes in the Ca^2+^ homeostasis and strong upregulation of antioxidant enzymes [48, 97]. Moreover, unique expression pattern of cold-shock proteins can exert tissue protection during hibernation. Cold-inducible RNA-binding proteins, so-called CIRP, have been reported by Horii et al. [48] in the myocardium of hibernating hamsters, showing that CIRP expression in the hamster heart is “regulated at the level of alternative splicing, which would permit a rapid increment of functional CIRP when entering hibernation” [48]. Interestingly, CIRP also targets cancer-associated mRNAs and can modulate inflammation [63], which might both be of importance to other organs’ protection during hibernation.

Beyond its protective effect against starvation, already decades ago it has been discovered that hibernation reduces the risk for radiation damage [73]. During hibernation, a remarkable resistance against radiation damage has been observed which could be helpful to counteract the radiation load during spaceflights [14, 116]. Hypothermic conditions in which core body temperature decreases well below 37 °C provide some protection against radiation-induced damage [14, 62] independent of whether hypothermia is applied before, during, or shortly after irradiation of cultured cells [62]. It was hypothesized that radioprotection during torpor may be the result of tissue hypoxia due to vasoconstriction [116] and decreased free radical production [21] as well as synergistically by reduced MR and a shift from glycolysis to lipolysis mimicking conditions during fasting [15].

## Occurrence of natural torpor among mammals

These potential benefits are contrasted by the fact that humans do not spontaneously enter torpor and no way is known to turn humans to torpor. Species known to enter torpor are widely distributed in most mammalian groups ranging from monotremes through metatheria and the majority of eutherian orders including primates [31, 43]. The ability for torpor and its absence may even be observed in systematically closely related species like for instance in rodents where mice frequently become torpid whereas in rats (*Rattus norvegicus*) torpor has never been observed. The reason for this species-specific expression of torpor behavior among mammals is presently not known, though these differences are taken advantage of in experimental settings, respectively [91].

Hibernation with multiday torpor bouts is a seasonal behavior which is driven by an endogenous circannual clock. The persistence of this behavior even under constant light and temperature conditions over several years was the first proof of the existence of a circannual clock in animals [81]. Also, daily torpor may be a seasonal behavior, e.g., in mouse lemurs or Djungarian hamsters [44, 77] or it can occur at any time of the year as a facultative behavior in response to food shortage and/or moderate cold exposure, i.e., in several species of small mammals like mice or small marsupials [22, 32, 52, 53].

**Fig. 3:**
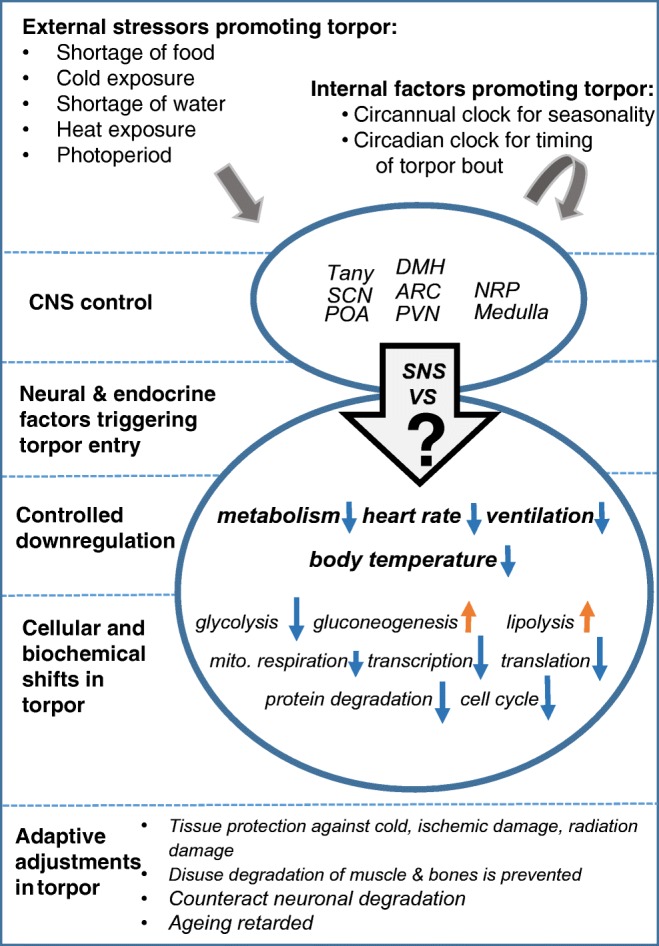
Summary of physiological changes during torpor in mammals. The central control of torpor includes activity of several brain areas mainly located in the hypothalamus. POA, the preoptic area, plays an important role in control of body temperature and its adjustment to lower levels in torpor. SCN, the suprachiasmatic nucleus, is controlling circadian rhythms and thus the timing of torpor bouts. PVN, the paraventricular nucleus, is involved in the control of food intake and response to environmental change. ARC, the arcuate nucleus, plays a leading role in homeostasis and contains specific neurons related to the control of feeding and metabolism. DMH, the dorsomedial hypothalamus, is involved in the control of feeding, body weight regulation, and circadian activity, and tightly linked with other neuronal areas related to these functions. NRP, the nucleus raphe pallidus, is tightly linked with hypothalamic neuronal areas, e.g., the PVN, and is involved in control of body temperature, heart rate, and ventilation. Tany, tanycites, are ependymal cells in the third ventricle transferring chemical signal from the cerebrospinal fluid to the brain neurons. Their ability to control thyroid hormone metabolism may be of significance for the expression of torpor behavior. The medulla contains several groups of neurons which are essential for most autonomic functions including ventilation and heart rate which undergo major changes during torpor. For further details of physiological, biochemical, and cellular adjustments during torpor, see text

All results obtained on physiological, biochemical, and endocrine changes during torpor are based on the observation of spontaneous occurrence of torpor [85]. Generally, environmental temperature and nutritional conditions are important factors enhancing torpid states. However, the trigger actually inducing torpor, either exogenous or endogenous, is not known (Fig. 3). Many different species can undergo torpor, either in the hottest desert region [38] or in the wintery North, so at every longitude and latitude and in all seasons. From an evolutionary point of view, torpor represents a strategy to cope with adverse environmental situations (“stressors”) as are cold or heat and dryness and lack of food availability. Thus, torpor is not restricted to hibernation but also may occur in tropical or subtropical habitat (e.g., the Malagassian lemurs [77]) to survive periods of dryness or in the case of daily torpor to safe energy.

This high capacity for metabolic control and changes in energy expenditure is an awesome example for “phenotypic plasticity.” However, it is an open question to which extent humans are capable of a similar phenotypic plasticity. At least, some phenotypic plasticity is seen in premature born humans [93], as well as in the protective mechanism of organs (e.g., brain, liver, kidney) to overcome damage, malperfusion, and malnutritional states by the principle of “preconditioning” (see below).

## Approaches to induce torpor in non-hibernating mammals

Although the mechanisms of torpor induction still are not known, some environmental factors involved in onset of seasonal torpor in hibernation have been identified, i.e., food shortage, temperature, and diurnal light and sleep cycles (Fig. 3). In addition, physiological readiness of the neuro-endocrine system is required [52]. Experimental modification of these factors revealed their significance but their action in concert still is a mystery.

In accord with the findings that food availability is one of the factors influencing onset of torpor, injection of leptin, mimicking large fat stores, reduced the duration and depth of daily torpor bouts [33, 34], while ghrelin injections, mimicking the need for food intake, enhance torpor occurrence in mice [35].

This underlines the significance of CNS and endocrine factors involved for the control of food intake for seasonal acclimation and the disposition to enter torpor (for review, see [52]). New findings in bears indicate that large mammals seem to “cycle” into a torpid state over weeks by slow but incrementally downregulated MR, with intermittent increases of MR [27].

Three parts of the nervous system have been identified to play a substantial role for control of MR and body temperature:

- The hypothalamus coordinating the majority of autonomic responses [46, 49, 80, 101]
- The *Raphe pallidus* nucleus in the brain stem which receives afferent inputs from several hypothalamic areas and projects to the spinal cord [13, 72]. It participates in the control of blood pressure, thermoregulatory heat production in brown fat, activity of heart, skeletal muscle, and smooth muscles, and, supposedly the thyroid and liver
- The sympathetic nervous system (SNS) is linking hypothalamic as well as extrahypothalamic areas and is strongly involved in the control of blood flow, ventilation and MR of peripheral organs, in torpor initiation, torpor maintenance as well as in arousal [7, 102].

Several attempts have been made to induce a torpor-like state in non-hibernators using drugs [5, 6]. Until now, the use of H_2_S, 5′-AMP and metamizol (an inhibitor of T4 deiodinization) and thyronamine was shown to be successful in reducing physiological parameters in mice and Djungarian hamsters [4, 8]. These are species which show torpor spontaneously as well, whereas other species did not respond in a similar way. Overall, the present findings do not give a clear picture about drugs reducing MR and changing metabolic pathways which could mimic natural torpor [5, 6].

## Human hibernation: an achievable goal?

Hibernation, torpor, and other hypometabolic states are widespread in mammals (and other vertebrates), in small (less than 10 kg) as well as in large animals (i.e., bear, deer). Up to now no unique factors or genes or neuro-endocrine properties have been identified for this state, suggesting that also humans have the potential for this physiological state. Hypometabolism in mammalian fetuses at normothermic conditions may provide a model for torpor in humans.

While small mammals, e.g., the Djungarian hamster, dormouse, and ground squirrel, reduce their temperature almost to the level of ambient temperature and need up to several hours for arousal from the torpid state, in larger mammals including bear and deer, body temperature remains well above 30 °C, but as shown for the brown bear, metabolism is reduced to 25% of summer activity [114]. They do not interrupt torpor by repeated arousals and torpor can be terminated within a short period of time. “High temperature torpor” (hypometabolism) is the preferred scheme for humans, because it is maintained continuously without energetically costly interruptions [20].

Humans, like other mammals, lower their metabolic rate and body temperature during sleep. Most pronounced changes were observed in Australian natives who were adapted to sleep without cover at low temperatures at night [39]. Pronounced depressions were also observed being associated with the chronic fatigue syndrome [75] or during deep meditation [45, 109]. The metabolic depression observed in these cases is much less than would be expected during a torpor bout. It demonstrates the potential for MR reduction in humans, but it is still uncertain whether and how these observations compare with natural torpor.

Analogies exist between the sequence of events occurring during entrance into torpor and adaptations to hypoxia [36] as to the diving reflex in aquatic mammals and humans [61, 79] or in human fetuses being exposed to birth asphyxia [93]. New observations seen in bears guide understanding the pattern how induction is occurring [27]. Moreover, preconditioning—a condition of non-damaging stimulus to promote a higher (transient, permanent) tolerance of tissues to a subsequent more severe/longer lasting challenge—seems also to work in humans and has strong parallels to metabolic changes in hibernating animals [18, 57, 58, 70, 84, 98, 108]. The (pre-)conditioning is furthermore one of the most efficient tools in biology to adapt to new environmental challenges on the cell organelles’ metabolic level and it seems to be effective in humans as well. Transient metabolic shutdown of organ metabolism, e.g., of the kidney, in the course of severe inflammatory disease and shock conditions is considered a state similar to hibernation [71]. For preconditioning, these benefits are regulated through the (i) preservation of energy in the cell, and hereby the (ii) preservation of cell homeostasis, and are linked to a distinct set of genes of which the expression was dampened in response to the preconditioning procedure. In particular, those genes affect glucose metabolism, protein turnover, and cell cycle [2].

Altogether, these effects “mimic hibernation and hypoxia tolerance suggesting the existence of a conserved endogenous genomic programme of physiological adaptations to oxygen limitation that improve survival” [98]. Since preconditioning can be achieved also by pharmacological interventions or hypoxic conditions—which have all shown their efficacy—these observations and conditions may help enable the first steps toward torpor in humans [41, 87]. The next steps have to be based on deeper knowledge of neuro-endocrine changes inducing torpor. The road map on the way transferring astronauts to torpor or a torpor-like state will include induction of torpor on animals which normally do not develop extensive MR reduction (e.g., rats, pigs). In parallel however—and because of the yet unknown risks of torpor in humans—the endeavor of manned mission to outer space needs to be accompanied also by technical developments, either in the spacecraft propulsion or spacious habitat design, including the implementation of artificial gravity. Moreover, the continuous increase in knowledge on the mitigation of the health risks under conditions of space environment will have to accompany and also help the new and game-changing venues enabling hibernation of a space crew.
